# AFFECTIVE TRAJECTORIES: RISK OF DEMENTIA AND UNDERLYING STRUCTURAL BRAIN VARIABLES IN OLDER WOMEN

**DOI:** 10.1093/geroni/igac059.913

**Published:** 2022-12-20

**Authors:** Andrew Petkus, Xinhui Wang, Susan Resnick, Daniel Beavers, Mark Espeland, Joshua Millstein, Margaret Gatz, Jiu-Chiuan Chen

**Affiliations:** University of Southern California, LOS ANGELES, California, United States; University of Southern California, Los Angeles, California, United States; National Institute on Aging, Baltimore, Maryland, United States; Wake Forest School of Medicine, Winston-Salem, North Carolina, United States; Wake Forest School of Medicine, Winston Salem, North Carolina, United States; University of Southern California, Los Angeles, California, United States; University of Southern California, Los Angeles, California, United States; University of Southern California, Los Angeles, California, United States

## Abstract

Understanding how trajectories of positive and negative affect relate to dementia risk and underlying structural brain variables is important for dementia prevention. We examined associations between annually assessed Positive and Negative Affect Scale subscales and dementia risk (2000-18) among cognitively-intact community-dwelling women (N=948; aged 72.9±3.7) from the Women’s Health Initiative Study of Cognitive Aging (years 2000-2010) and Magnetic Resonance Imaging Study (2005-2006). Joint latent class mixture models were constructed to identify latent classes of women with similar trajectories of affect and dementia risk over time. Multinomial and logistic regressions examined whether structural MRI measures predicted latent class membership (adjusted for sociodemographic, lifestyle, clinical characteristics, and intracranial volume). Two latent classes of positive affect (high stable:88% and decreasing:12%) and four classes of negative affect (Minimal stable:75%; high stable:4%; emerging:12%; moderate decreasing:9%) were identified. With the high stable trajectory as referent, women with decreasing positive affect were more likely to develop dementia (HR=4.33;p<.001). The odds of being classified as this high-dementia risk group were increased among women with more (per SD) global small vessel ischemic disease (SVID;OR=1.42;p<.001), deep white matter SVID (OR=1.93;p<.001), and smaller parahippocampal volumes (OR=1.41;p=.016). For negative affect, with minimal stable negative affect as referent, women with smaller hippocampal volumes were more likely to be classified as having moderate decreasing negative affect (OR=1.45;p=.024) while emerging negative affect was associated with higher dementia risk (HR=2.00;p=.014). These findings highlight the importance of changes in affect in later-life with dementia risk and potential underlying role of cerebrovascular disease and medial temporal lobe structures.

